# Modeling and Simulation of Tin Sulfide (SnS)-Based Solar Cell Using ZnO as Transparent Conductive Oxide (TCO) and NiO as Hole Transport Layer (HTL)

**DOI:** 10.3390/mi13122073

**Published:** 2022-11-25

**Authors:** Ahmad Umar, Pooja Tiwari, Sadanand, Vaibhava Srivastava, Pooja Lohia, Dilip Kumar Dwivedi, Hussam Qasem, Sheikh Akbar, Hassan Algadi, Sotirios Baskoutas

**Affiliations:** 1 Department of Chemistry, College of Science and Arts, Promising Centre for Sensors and Electronic Devices (PCSED), Najran University, Najran 11001, Saudi Arabia; 2 Department of Materials Science and Engineering, The Ohio State University, Columbus, OH 43210, USA; 3 Department of Electronics and Communication Engineering, Madan Mohan Malviya University of Technology, Gorakhpur 273010, India; 4 Department of Applied Sciences, Galgotias College of Engineering and Technology, Greater Noida 201306, India; 5 Photonics and Photovoltaic Research Lab, Department of Physics and Material Science, Madan Mohan Malviya University of Technology, Gorakhpur 273010, India; 6 National Centre for Renewable Energy, King Abdulaziz City for Science and Technology (KACST), Riyadh 11442, Saudi Arabia; 7 Department of Electrical Engineering, College of Engineering, Najran University, Najran 11001, Saudi Arabia; 8 Department of Materials Science, University of Patras, 26500 Patras, Greece

**Keywords:** SnS, heterojunction solar cell, CdS

## Abstract

This paper describes the simulation by Solar Cell Capacitance Simulator-1D (SCAPS-1D) software of ZnO/CdS/SnS/NiO/Au solar cells, in which zinc oxide (ZnO) is used as transparent conductive oxide (TCO) and nickel oxide (NiO) is used as a hole transport layer (HTL). The effects of absorber layer (SnS) thickness, carrier concentration, SnS defect density, NiO HTL, ZnO TCO, electron affinity and work function on cell performance have been evaluated. The effect of interface defect density of SnS/CdS on the performance of the heterojunction solar cell is also analysed. As the results indicate, a maximum power conversion efficiency of 26.92% was obtained.

## 1. Introduction

Global energy consumption is rising as a result of population growth and a wide use of digital substances in practically every aspect of our lives. The major source of energy is fossil fuels [1] which is not a long-term energy source. The burning of fossil fuels emits a large amount of carbon dioxide [2]. Furthermore, fossil fuels are not a sustainable source. For these reasons, the energy sector is expanding but shifting to sustainable sources of energy as the optimum choice. Solar cells can be a viable substitute for fossil fuels. Many researchers have recently concentrated on highly efficient polymer and perovskite cells [3,4], but these solar cells suffer from instability which limits their long-term application areas. As a result, the thin-film solar cell may be used in place of polymer and perovskite solar cells.

Tin monosulphide (SnS) has been developed as a significant absorbent layer in thin-film solar cells having various advantages such as a high absorption coefficient that allows SnS to absorb a considerable portion of the spectrum. SnS is an Earth-abundant material which can be manufactured at low cost [5,6,7,8,9]. The energy gap at the absorber and back contact interface has a significant impact on the performance of the cell. This energy gap can be reduced by using a small resistive back contact with higher work function (WF). High WF transition metal oxides (TMOs) such as MoO_X_ [10,11,12]_,_ V_2_O_5_ [13], NiO [14] and WO_3_ [15] were used as HTLs in several solar cells for performance improvement. NiO is a highly useful HTL within all TMOs due to its wide bandgap (3.5–3.8 eV) and high WF (5 eV). Polycrystalline CdS is used as a window layer, having many features such as high absorption coefficient, electron affinity, low resistivity, high optical transmittance, wide bandgap and good electrical properties that are suitable for solar cell fabrication and applications as well. TCO in a solar cell is usually indium tin oxide (ITO) or fluorine-doped tin oxide (FTO) which are quite expensive, and ITO has stability limitations. ZnO is used as TCO, having a wide bandgap of 3.37 eV, excellent electrical and optical properties, and it is Earth abundant [16,17]. Furthermore, different parameters such as carrier concentration, SnS layer thickness and defects have a considerable impact on the performance of the cell [18,19,20,21].

In this study, a new highly efficient structure of ZnO/CdS/SnS/NiO/Au of SnS-based solar cells is designed. SCAPS 1-D software is used to perform a numerical study of the developed cell. The influence of various parameters of ZnO, SnS and NiO HTL on the performance of the solar cell has been studied. The improved solar cell achieved a maximum efficiency of 26.92%.

## 2. Modelling and Simulation

Simulation software of solar cells is designed to solve semiconductor material property equations. SCAPS 1-D software is much more popular than other simulation software due to its ability to simulate up to seven layers of structure [22,23]. SCAPS 1-D simulator is used in the modelling of ZnO/CdS/SnS/NiO/Au heterostructure cells. It includes the following equations for measurements of J-V, C-V, C-F properties and quantum efficiency of the solar cell.

Poisson’s equation: (∂^2^ Ψ/∂x^2^) + (q/ε) [ p(x) − n(x) + N_D_ − N_A_ + ρ_p_ − ρ_n_= 0, (1)

Hole continuity equation: (1/q) (∂J_p_/∂x) = G_op_ − R(x), (2)

Electron continuity equation: (1/q) (∂Jn/∂x) = −Gop + R(x),(3)

where ε = dielectric constant, q = charge of the electron, N_A_ = acceptor density, N_D_ = donor density, Ψ = electrostatic potential, Jp = current density due to holes, Jn = current density due to electrons, G_op_ = carrier generation rate, R = total recombination rate, ρ_p_ and ρ_n_ = hole and electron distribution, respectively.

The semiconducting material’s holes and electron charge carrier characteristics are represented by the drift and diffusion equations [24] given below:

J_p_ = −(μ_p_ p)/q (∂E_Fp_/∂x), (4)

J_n_ = −(μ_n_ n)/q (∂E_Fn_/∂x), (5)

where μ_p_ and μ_n_ = holes and electron mobility, respectively, E_Fp_ and E_Fn_ = p-type and n-type fermi level, respectively.

## 3. Solar Cell Structure and Material Properties

**Figure 1:**
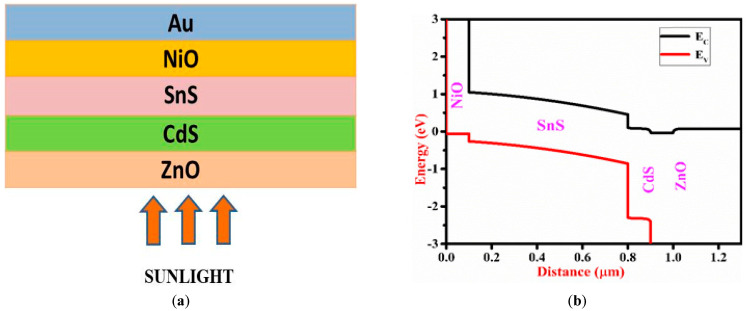
(**a**) Schematic structure and (**b**) energy band diagram of the proposed solar cell.

The schematic structure and energy band diagram of the proposed ZnO/CdS/SnS/NiO/Au cell are shown In Figure 1a,b with ZnO serving as TCO, CdS as the window layer, SnS as the absorber layer, NiO as HTL and Au as back metal contact. ZnO is an n-type semiconductor used as the substitute of ITO and FTO because of its excellent electrical and optical properties. Figure 1b shows that the conduction band of SnS is smaller than that of CdS and the conduction band offset (CBO) between them is also very low. Thus, electrons can flow easily from SnS to ZnO through CdS. The valence band of NiO is higher than that of SnS and the valence band offset (VBO) between them is smaller. Furthermore, CBO between SnS and NiO is relatively high, stopping electrons from entering the back electrode. For benchmarking purposes, we have used a ZnO/CdS/CdTe/SnS/Ni device structure [25]. The physical parameters used in the modelling of ZnO/CdS/SnS/NiO are stated in Table 1 and interface parameters of SnS/CdS are listed in Table 2.

## 4. Results and Discussion

**Figure 2:**
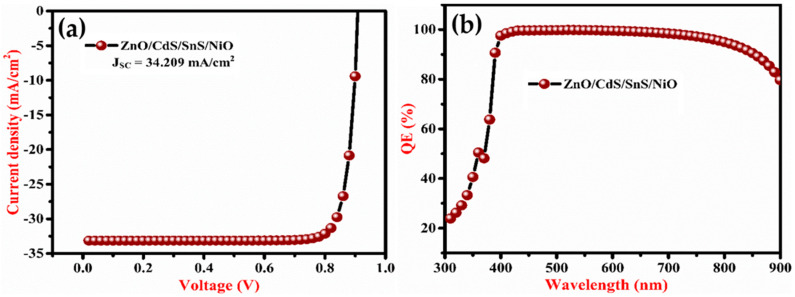
(**a**) J-V curve and (**b**) quantum efficiency of the proposed heterojunction solar cell.

The heterostructure ZnO/CdS/SnS solar cells have V_OC_, J_SC_, FF and PCE of 0.4732 V, 33.739 mA/cm^2^, 71.79% and 11.46%, respectively. Figure 2a,b display the J-V characteristics and quantum efficiency (QE) of the heterostructure ZnO/CdS/SnS/NiO/Au solar cell. The improved solar cell achieved V_OC_, J_SC_, FF and PCE of 0.9048 V, 34.209 mA/cm^2^, 86.97% and 26.92%, respectively. After including NiO and other materials, the QE of the solar cell increases from the wavelength of 600-900 nm as shown in Figure 2(b). This enhancement of QE shows the reduction in surface carrier recombination at SnS that demonstrated the improvement of PCE after employing NiO as the HTL. The PCE of the enhanced cell is increased below the wavelength of 400 nm.

### 4.1. Impact of Carrier Concentration and Thickness of NiO and ZnO

The cell performance was studied in terms of carrier concentration and layer thickness. The thickness of NiO and ZnO was varied from 100 to 12,000 nm and carrier concentration was varied from 10^14^ to 10^21^ cm^−3^. The V_OC_ is nearly independent of both carrier concentration and NiO thickness. The J_SC_ varies significantly depending on carrier concentration and thickness. At a NiO carrier concentration of 10^18^ cm^−3^ and thickness of 250 nm, the optimal J_SC_ of 34.20 mA/cm^2^ was obtained. The FF and PCE increase with an increase in carrier concentration but remain almost constant with increasing thickness. The series resistance decreases as the carrier concentration increases due to which FF and PCE rise [10]. At a NiO carrier concentration of 10^21^ cm^−3^ and thickness of 250 nm, the maximum PCE of 26.92% is recorded.

The thickness of ZnO does not have any effect on V_OC_ but fluctuates with carrier concentration and achieves its maximum value of 0.9048 V at 10^17^ cm^−3^. The J_SC_ worked similarly to the V_OC_ and gave an optimal value of J_SC_ 34.20 mA/cm^2^ at 10^17^ cm^−3^ carrier concentration. The increase in ZnO carrier concentration induced band bending resulting in a small rise in J_SC_. The FF had a reducing nature with increasing thickness of ZnO up to 10^17^ cm^−3^ because of increasing series resistance. Maximum efficiency of 26.92% is recorded at carrier concentration and thickness of 10^17^ cm^−3^ and 100 nm, respectively.

### 4.2. Impact of SnS/CdS Interface Defect Density

The influences of SnS/CdS interface defect density (IDD) on the performance of the proposed solar cell have been analysed. The SnS/CdS defect interface can increase the series resistance and carrier trapping of the cell. Due to the increase in carrier recombination rate at the interface, V_OC_ decreases with IDD but IDD has no impact on J_SC_ until 10^17^ cm^2^. J_SC_ increases from 19.48 to 28.25 mA/cm^2^ when the width of SnS layer increases from 200 to 1200 nm. When IDD increases from 10^11^ to 10^18^ cm^2^, FF decreases rapidly from 84.9% to 54.8% due to an increase in the series resistance of the cell, indicating that high defect density at SnS/CdS is responsible for high series resistance. A significant decrease in PCE was also seen with increasing IDD. An interface defect is one of the reasons for lower PCE. Defects in the interfacial layer density are induced in the cell as a result of structural changes in the materials produced during the fabrication process.

### 4.3. Impact of Defect Density and Thickness of Absorber Layer SnS

**Figure 3:**
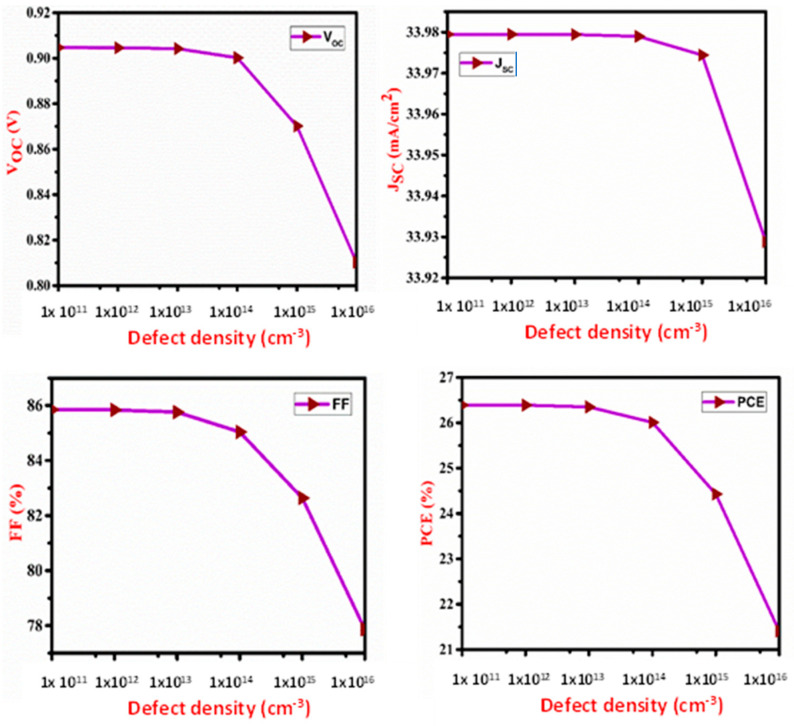
Impact of defect density of SnS layer on V_OC_, J_SC_, FF and PCE of the proposed cell.

**Figure 4:**
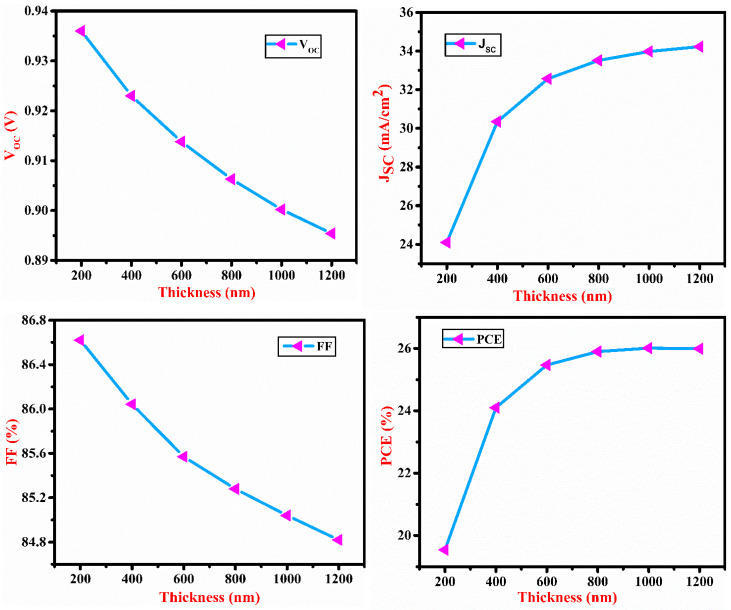
Impact of SnS layer thickness on V_OC_, J_SC_, FF and PCE of the proposed cell.

The influence of absorber layer defect density and thickness is depicted in Figure 3 and Figure 4, respectively. The defect is caused by displacement and surface defects. Defects serve in carrier recombination, reducing mobility and carrier lifetime. The Shockley–Read–Hall (SRH) process modulates the recombination rate of SnS at higher defect density.

The absorber layer thickness and defect density varied from 100 to 1200 nm and from 10^11^ to 10^16^ cm^−3^, respectively. V_OC_ decreases with an increase in thickness and defect density of SnS but there is no effect of defect density on J_SC_ below 10^15^ cm^−3^ due to an increase in carrier recombination. Due to the high absorption of increased wavelength photons in the layer, J_SC_ increases up to a certain level with an increase in the thickness of SnS. After that, it saturates due to light absorption saturation. PCE is increased with SnS thickness as the J_SC_ increases and saturates at 1200 nm due to the light absorption saturation. The fill factor shows the same nature as V_OC_. J_SC_, V_OC_ and FF are all associated with PCE. At defect density of 10^11^ cm^−3^ and thickness of the SnS layer of 1000 nm, the maximum PCE of 26.92% is recorded.

### 4.4. Impact of Electron Affinity and Back Contact Metal Work Function of Absorber Layer SnS

**Figure 5:**
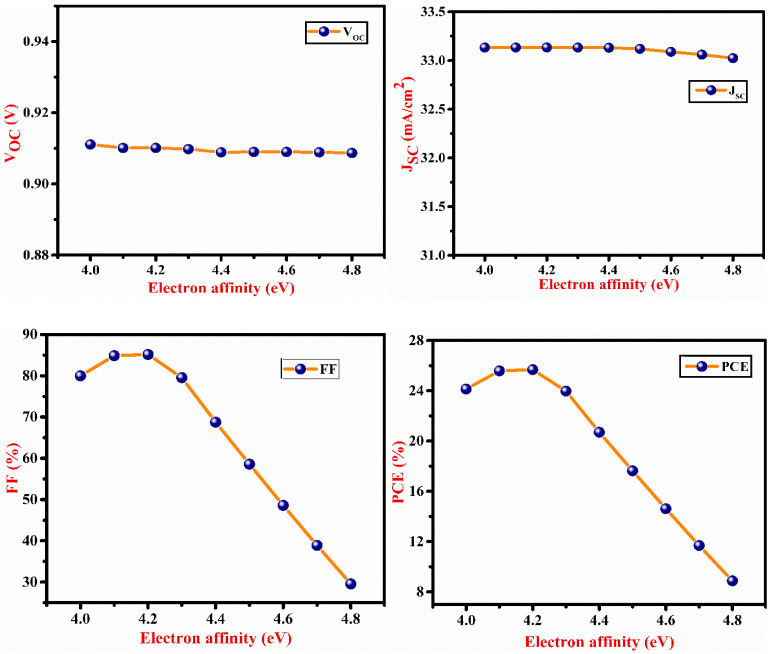
Impact of the electron affinity of SnS layer on V_OC_, J_SC_, FF and PCE of the proposed cell.

**Figure 6:**
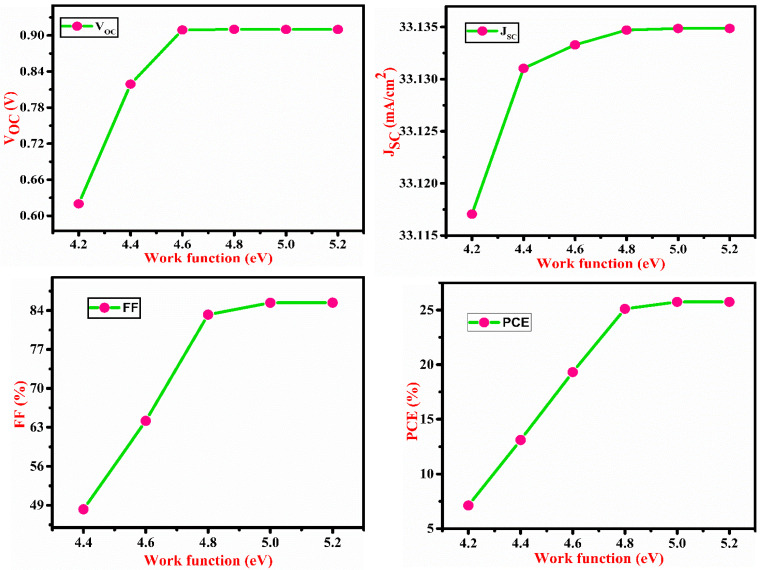
Impact of back contact metal work function on V_OC_, J_SC_, FF and PCE of the proposed cell.

Figure 5 depicts the effect of SnS electron affinity (EA). The maximum performance of the cell was obtained at SnS EA of 4.3 eV. Due to a decrease in FF, PCE falls with EA > 4.3 eV due to a mismatch of energy level between the SnS and CdS layer for electron transport as shown in Figure 5. V_OC_ does not show any significant change with affinity due to insufficient carriers. The effect of back contact metal work function (WF) is shown in Figure 6. As the WF increases, J_SC_, V_OC_, FF and PCE also increase up to a certain WF level. This indicates that when WF rises, the majority carrier barrier height reduces. As a result, PCE rises with WF till 5 eV, and after 5 eV, PCE saturates. Thus, WF has a significant influence on the performance of the solar cell. Proper metal contact is necessary to achieve high efficiency. Table 3 shows a comparison of the physical parameters of various simulated device structures. Our findings seem to have a satisfying consistency with those that have already been published.

## 5. Conclusions

The performance of the cell structure ZnO/CdS/SnS/NiO/Au has been investigated using SCAPS 1-D software. It is shown that NiO is a suitable HTL material with the ability to improve the efficiency of SnS-based solar cells. ZnO can be a good replacement for ITO and FTO in the fabrication of low-cost and highly efficient SnS-based solar cells. At ZnO and NiO carrier concentrations of 10^17^ and 10^21^ cm^−3^, respectively, and thicknesses of 100 and 250 nm, respectively, the maximum PCE of 26.92% is recorded. The thickness and defect density of the SnS layer were also investigated. As the SnS thickness increases, the PCE of the cell also increases. The maximum PCE of the proposed solar cell is recorded at thickness and defect density of 1000 nm and 10^11^ cm^−3^. The present work might give an insight into the modelling and development of the high performance of SnS-based solar cells.

## Figures and Tables

**Table 1:** Parameters of ZnO [26], CdS [27], SnS [28] and NiO [14] used in the simulation of proposed solar cell.

Parameters	ZnO	CdS	SnS	NiO
Thickness (nm)	100	100	1000	250
Bandgap (eV)	3.37	2.4	1.31	3.8
Electron affinity (eV)	4.5	4.5	4.3	1.46
Dielectric permittivity (relative)	9	10	13	10
CB effective density of states (cm^−3^)	2.2 × 10^18^	2.2 × 10^18^	1.18 × 10^18^	2.8 × 10^19^
VB effective density of states (cm^−3^)	1.8 × 10^18^	1.9 × 10^19^	4.76 × 10^18^	1 × 10^18^
Electron mobility (cm^2^/V_S_)	100	350	130	12
Hole mobility (cm^2^/V_S_)	25	25	4.3	2.8
Shallow uniform donor density N_d_ (cm^−3^)	1 × 10^17^	1 × 10^17^	1 × 10^7^	0
Shallow uniform acceptor density N_a_ (cm^−3^)	0	0	1 × 10^15^	1 × 10^21^
Electron thermal velocity (cm/s)	1 × 10^7^	1 × 10^7^	1 × 10^7^	1 × 10^7^
Hole thermal velocity (cm/s)	1 × 10^7^	1 × 10^7^	1 × 10^7^	1 × 10^7^
Defect density (cm^−3^)	1 × 10^14^	1 × 10^14^	1 × 10^11^	1 × 10^14^
Radiative recombination coefficient (cm^3^/s)	2.3 × 10^−9^	2.3 × 10^−9^	2.3 × 10^−9^	2.3 × 10^−9^
Absorption coefficient (cm^−1^)	SCAPS	1 × 10^5^	1 × 10^5^	1 × 10^5^

**Table 2:** Interface parameters used in the simulation of the proposed solar cell.

Parameters	SnS/CdS Interface [28]
Defect type	Neutral
Capture cross-section electrons (cm^2^)	1 × 10^−19^
Capture cross-section holes (cm^2^)	1 × 10^−19^
Defect energy level E_t_	Above the highest E_v_
Energy with respect to a reference (eV)	0.06
Total density (cm^−2^)	1 × 10^10^

**Table 3:** Comparison of previously reported results to proposed work.

Structures	V_OC_ V	J_SC_ mA/cm^2^	FF %	PCE %	References
ITO/CeO_2_/SnS/NiO/Mo (simulated ITO)	0.890	32.67	86.19	25.06	[14]
ITO/CeO_2_/SnS/Spiro-OMeTAD (simulated)	0.887	33.74	85.61	25.65	[28]
p-SnS/CdS/n-Zn MgO (simulated)	~0.7	38.54	83	~23	[29]
ZnO/CdS/SnS (simulated)	0.73	33.20	61.47	14.9%	[30]
ZnO/CdS/CdTe/SnS/Ni (simulated)	0.845	26.46	84.50	21.83	[24]
ZnO/CdS/SnS/NiO (simulated)	0.904	34.20	86.97	26.92	This paper

## Data Availability

All data is provided in the manuscript.
